# Supplementary figures and images for: Bile acid 7α-dehydroxylating bacteria accelerate injury-induced mucosal healing in the colon
Source: EMBO Mol Med. 2025 Mar 10;17(5):889–908. doi: 10.1038/s44321-025-00202-w (PMC12081655; doi:10.1038/s44321-025-00202-w)

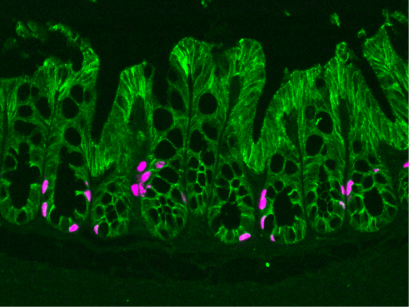

Supplement: Supplementary file 7 — Source data Fig. 1 [file 44321_2025_202_MOESM7_ESM.zip › Figure 1/1J/Fig1J OligoMM12.tif]

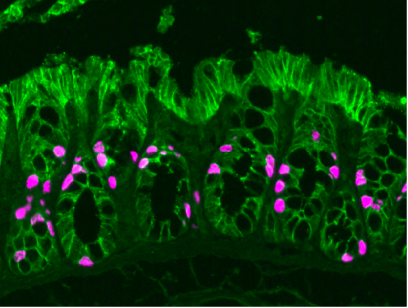

Supplement: Supplementary file 7 — Source data Fig. 1 [file 44321_2025_202_MOESM7_ESM.zip › Figure 1/1J/Fig1J OligoMM12 Cscindens.tif]

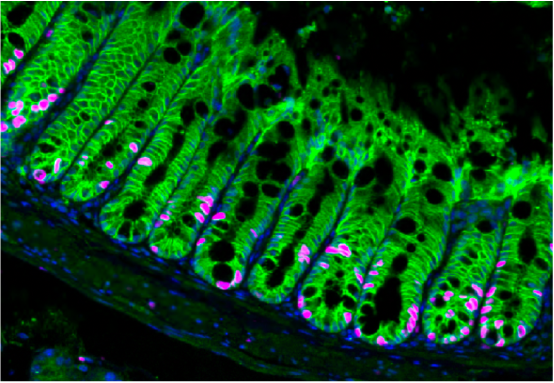

Supplement: Supplementary file 8 — Source data Fig. 2 [file 44321_2025_202_MOESM8_ESM.zip › Figure 2/2O/Fig2O SPF-van.tif]

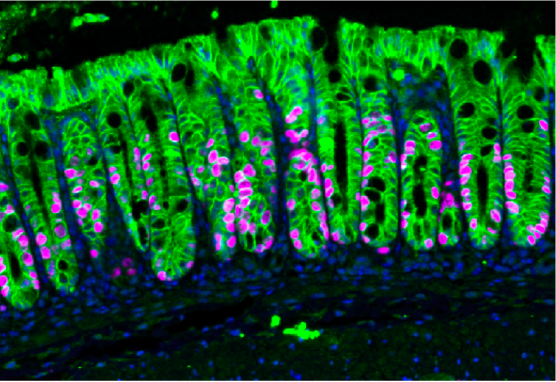

Supplement: Supplementary file 8 — Source data Fig. 2 [file 44321_2025_202_MOESM8_ESM.zip › Figure 2/2O/Fig2O SPF-van Cscindens.tif]

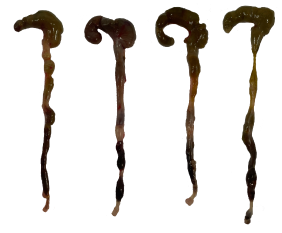

Supplement: Supplementary file 8 — Source data Fig. 2 [file 44321_2025_202_MOESM8_ESM.zip › Figure 2/2H/Fig2H SPF-van Cscindens.tif]

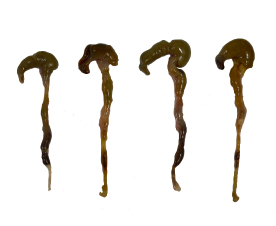

Supplement: Supplementary file 8 — Source data Fig. 2 [file 44321_2025_202_MOESM8_ESM.zip › Figure 2/2H/Fig2H SPF-van.tif]

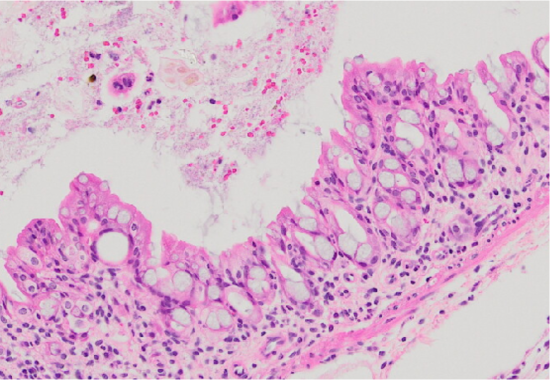

Supplement: Supplementary file 8 — Source data Fig. 2 [file 44321_2025_202_MOESM8_ESM.zip › Figure 2/2K/Fig2K SPF-van.tif]

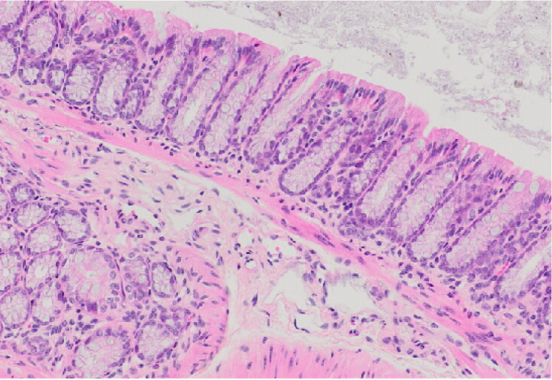

Supplement: Supplementary file 8 — Source data Fig. 2 [file 44321_2025_202_MOESM8_ESM.zip › Figure 2/2K/Fig2K SPF-van Cscindens.tif]

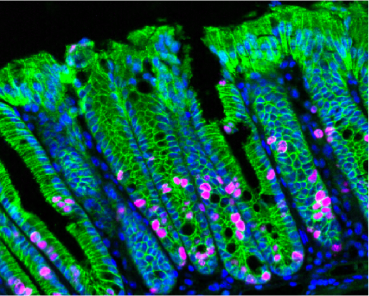

Supplement: Supplementary file 9 — Source data Fig. 3 [file 44321_2025_202_MOESM9_ESM.zip › Figure 3/3E/Fig3E Tgr5++ SPF-van.tif]

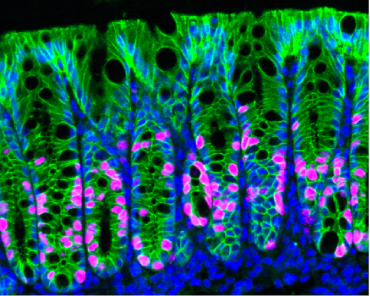

Supplement: Supplementary file 9 — Source data Fig. 3 [file 44321_2025_202_MOESM9_ESM.zip › Figure 3/3E/Fig3E Tgr5++ SPF-van Cscindens.tif]

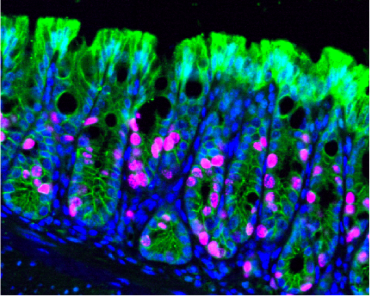

Supplement: Supplementary file 9 — Source data Fig. 3 [file 44321_2025_202_MOESM9_ESM.zip › Figure 3/3E/Fig3E Tgr5-- SPF-van Cscindens.tif]

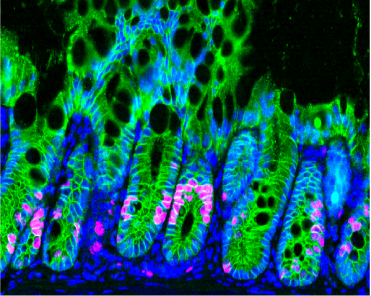

Supplement: Supplementary file 9 — Source data Fig. 3 [file 44321_2025_202_MOESM9_ESM.zip › Figure 3/3E/Fig3E Tgr5-- SPF-van.tif]

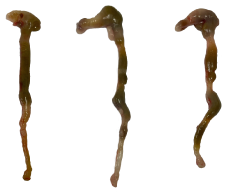

Supplement: Supplementary file 9 — Source data Fig. 3 [file 44321_2025_202_MOESM9_ESM.zip › Figure 3/3C/Fig3C Tgr5++ SPF-van.tif]

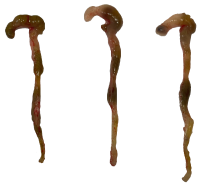

Supplement: Supplementary file 9 — Source data Fig. 3 [file 44321_2025_202_MOESM9_ESM.zip › Figure 3/3C/Fig3C Tgr5-- SPF-van.tif]

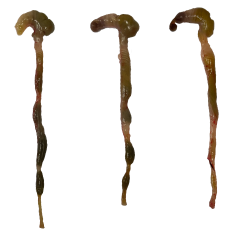

Supplement: Supplementary file 9 — Source data Fig. 3 [file 44321_2025_202_MOESM9_ESM.zip › Figure 3/3C/Fig3C Tgr5++ SPF-van Cscindens.tif]

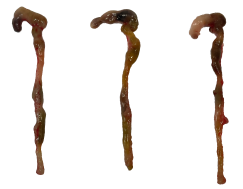

Supplement: Supplementary file 9 — Source data Fig. 3 [file 44321_2025_202_MOESM9_ESM.zip › Figure 3/3C/Fig3C Tgr5-- SPF-van Cscindens.tif]

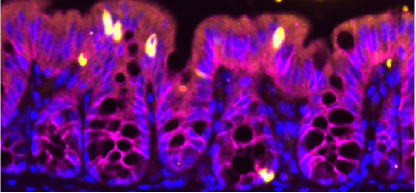

Supplement: Supplementary file 9 — Source data Fig. 3 [file 44321_2025_202_MOESM9_ESM.zip › Figure 3/3M/Fig3M Tgr5++ SPF-van.tif]

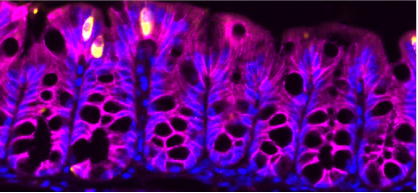

Supplement: Supplementary file 9 — Source data Fig. 3 [file 44321_2025_202_MOESM9_ESM.zip › Figure 3/3M/Fig3M Tgr5-- SPF-van.tif]

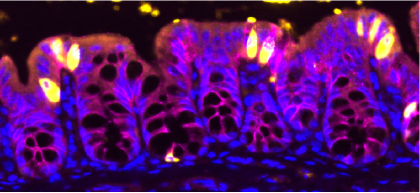

Supplement: Supplementary file 9 — Source data Fig. 3 [file 44321_2025_202_MOESM9_ESM.zip › Figure 3/3M/Fig3M Tgr5++ SPF-van Cscindens.tif]

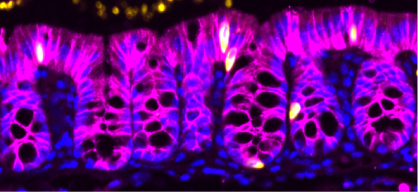

Supplement: Supplementary file 9 — Source data Fig. 3 [file 44321_2025_202_MOESM9_ESM.zip › Figure 3/3M/Fig3M Tgr5-- SPF-van Cscindens.tif]
