# Supplementary material for: Bile acid 7α-dehydroxylating bacteria accelerate injury-induced mucosal healing in the colon
Source: EMBO Mol Med. 2025 Mar 10;17(5):889–908. doi: 10.1038/s44321-025-00202-w (PMC12081655; doi:10.1038/s44321-025-00202-w)
Supplement: Supplementary file 11 — Expanded View Figures [file 44321_2025_202_MOESM11_ESM.pdf]

Expanded View Figures

Figure EV1. Stable colonization of Oligo-MM^{12} mice with *C. scindens* modulates the BA composition and promotes proliferation of colonic stem cells under basal conditions.

(**A**) Schematic illustrating the 7α-dehydroxylation reaction at carbon 7 (highlighted in red) of CDCA to form LCA. (**B**) Violin plots showing the amount of the indicated BA species in the feces of 8-week-old male Oligo-MM^{12} mice gavaged with live *C. scindens* (10^7 CFU – Oligo-MM^{12} + *C. scindens*) or PBS (control – Oligo-MM^{12}) under basal conditions ($n = 7$/group) (For LCA (nmol/g): Oligo-MM^{12} vs Oligo-MM^{12} + *C. scindens* $P < 0.0001$). (**C**) Dot plots representing the BA proportion and the z score in stools of mice in (**B**). The dot color represents the z-score for each BA species, whereas the dot size represents the proportion of each BA species over the total BA amount per mouse. 7α-dehydroxylated BAs are highlighted in red. Ctrl = Oligo-MM^{12} and *Cs* = Oligo-MM^{12} + *C. scindens*. (**D**) Plasma BA composition of mice in (**B**). BA data are normalized to the total amount of BAs. 7α-dehydroxylated BAs are in red. (**E**, **F**) Concentrations (nM) of the indicated BAs and secondary (SBA)-to-primary (PBA) ratio in plasma of mice in (**B**). 7α-DH-ed: 7α-dehydroxylated BAs. (**G**) BA hydrophobicity index in plasma of mice in (**B**). (**H**, **I**) Relative mRNA levels of the indicated genes in liver (**H**) or ileum (**I**) of mice in (**B**). (**J**, **K**) Representative images (**J**) and quantification of EdU$^+$ cells per crypt (**K**) in the jejunum, ileum, and colon of mice in (**B**). Graphs represent mean ± SEM. *n* refers to biological replicates. *P* values (exact values) were calculated using 2-tailed Student's t test (**B**, **E**, **F**, **G**, **K**).

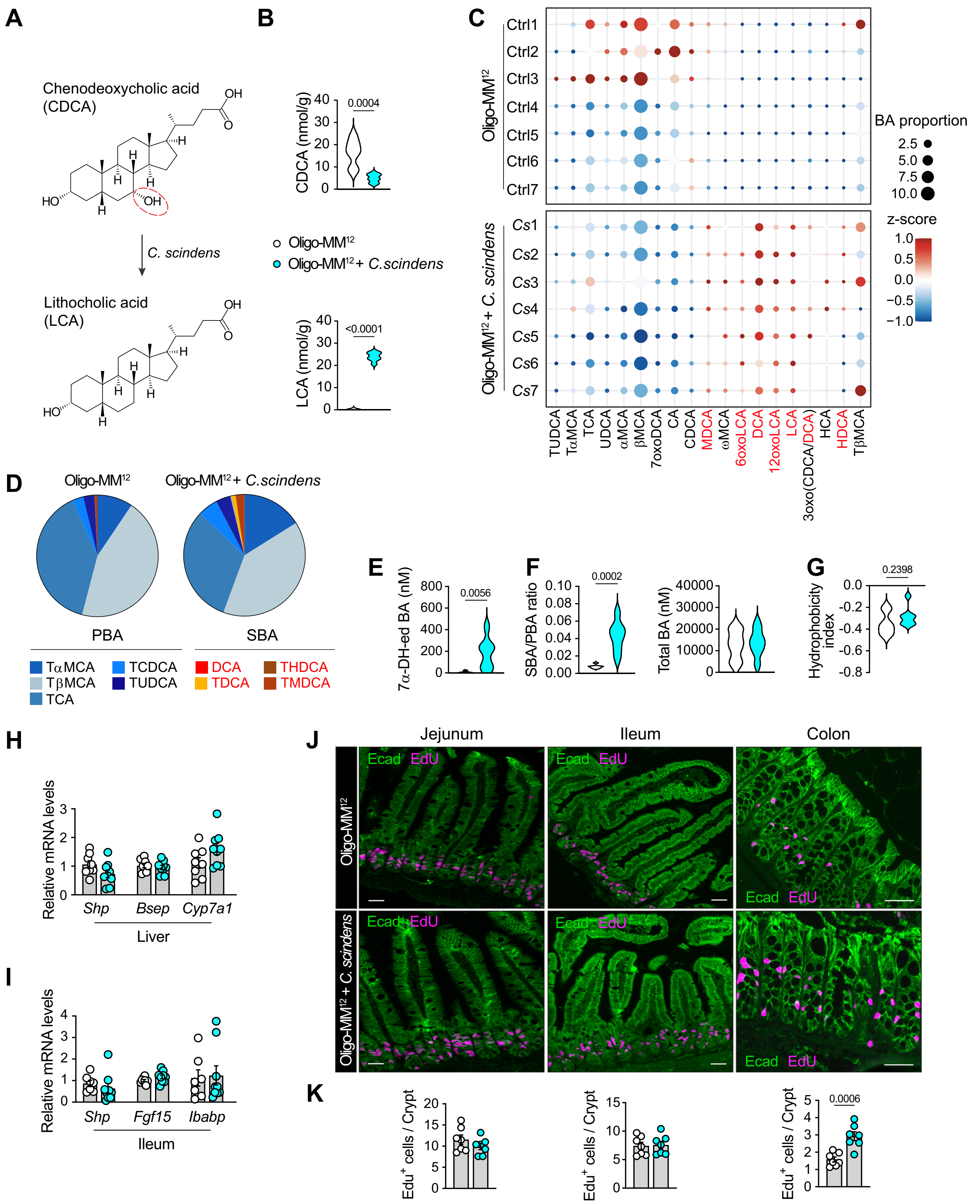
A
Chenodeoxycholic acid (CDCA)
C. scindens
Lithocholic acid (LCA)
B
CDCA (nmol/g)
0.0004
LCA (nmol/g)
<0.0001
Oligo-MM12
Oligo-MM12 + C.scindens
C
BA proportion
z-score
D
PBA
SBA
TαMCA TβMCA TCA TCDCA TUDCA
DCA TDCA THDCA TMDCA
E
7α-DH-ed BA (nM)
0.0056
F
SBA/PBA ratio
0.0002
Total BA (nM)
G
Hydrophobicity index
0.2398
H
Relative mRNA levels
Shp Bsep Cyp7a1
Liver
I
Relative mRNA levels
Shp Fgf15 Ibabp
Ileum
J
Jejunum
Ileum
Colon
Ecad EdU
Oligo-MM12 + C. scindens
K
Edu+ cells / Crypt
0.0006

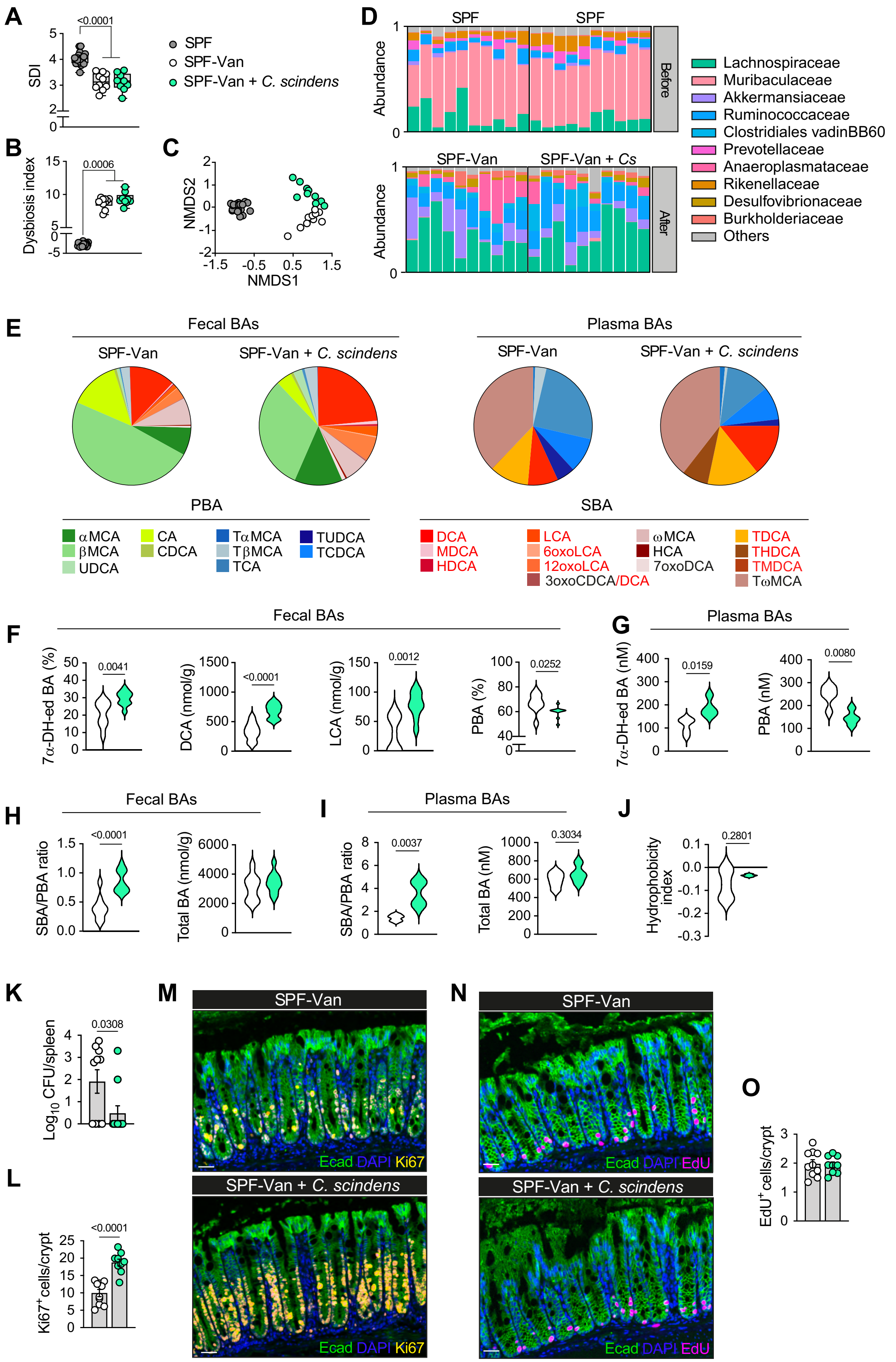

SPF
SPF-Van
SPF-Van + C. scindens
SDI
Dysbiosis index
NMDS1
NMDS2
Abundance
Before
After
Lachnospiraceae
Muribaculaceae
Akkermansiaceae
Ruminococcaceae
Clostridiales vadinBB60
Prevotellaceae
Anaeroplasmataceae
Rikenellaceae
Desulfovibrionaceae
Burkholderiaceae
Others
Fecal BAs
Plasma BAs
PBA
SBA
αMCA
βMCA
UDCA
CA
CDCA
TαMCA
TβMCA
TCA
TUDCA
TCDCA
DCA
MDCA
HDCA
LCA
6oxoLCA
12oxoLCA
3oxoCDCA/DCA
ωMCA
HCA
7oxoDCA
TDCA
THDCA
TMDCA
TωMCA
7α-DH-ed BA (%)
DCA (nmol/g)
LCA (nmol/g)
PBA (%)
7α-DH-ed BA (nM)
PBA (nM)
SBA/PBA ratio
Total BA (nmol/g)
Total BA (nM)
Hydrophobicity index
Log10 CFU/spleen
Ki67+ cells/crypt
EdU+ cells/crypt
Ecad DAPI Ki67
Ecad DAPI EdU

Figure EV2. Characterization of *C. scindens* colonization in SPF-Van mice.

(**A**) Shannon diversity index (SDI) showing the fecal bacterial community diversity of C57BL/6J mice (SPF) ($n = 19$) or preconditioned with vancomycin and gavaged daily for 5 days with live *C. scindens* (10^8 CFU – SPF-Van + *C. scindens*) ($n = 9$) or PBS vehicle (SPF-Van) ($n = 10$) (SPF vs all other groups $P < 0.0001$). (**B**) Gut dysbiosis index of mice in (**A**). (**C**) Non-metric multidimensional scaling (NMDS) of amplicon sequence variant (ASV) counts based on the Bray-Curtis dissimilarity of mice in (**A**). (**D**) Relative abundance of bacterial families representing more than 10% of total abundance of mice in (**A**). (**E**) BA composition in feces ($n = 9$ for SPF-Van and $n = 10$ for SPF-Van + *C. scindens*) and plasma ($n = 5$ for SPF-Van and $n = 4$ for SPF-Van + *C. scindens*), respectively. BA data are normalized to the total amount of BAs. 7α-dehydroxylated BAs are in red. (**F**) Violin plots showing BA amount (nmol/g of feces) or proportion of the indicated BAs to total BA fecal amount of mice in (**E**) (For DCA (nmol/g): SPF-Van vs SPF-Van + *C. scindens* $P < 0.0001$). (**G**) Violin plots showing the BA concentration (nM) of the indicated BAs in the plasma of mice in (**E**). (**H**) Secondary (SBA)-to-primary (PBA) BA ratio and total BA amount in feces of mice in (**E**) (For SBA/PBA ratio: SPF-Van vs SPF-Van + *C. scindens* $P < 0.0001$). (**I**) Secondary (SBA)-to-primary (PBA) BA ratio and total BA concentration in plasma of mice in (**E**). (**J**) BA hydrophobicity index in plasma of mice in (**E**). (**K**) Bacterial colony-forming units (CFU) in the spleen of 10-week-old male SPF-Van mice gavaged daily for 5 days with live *C. scindens* (10^8 CFU – SPF-Van + *C. scindens*) ($n = 11$) or PBS (control – SPF-Van) ($n = 10$) and subjected to a 7-day treatment with DSS (2.5% in drinking water) (Fig. 2F). (**L**, **M**) Quantification (**L**) and representative images (**M**) of Ki67$^+$ cells per crypt in the colon of 10-week-old male SPF-Van mice gavaged daily for 5 days with live *C. scindens* (10^8 CFU – SPF-Van + *C. scindens*) ($n = 9$) or vehicle (PBS – SPF-Van) ($n = 10$) (SPF-Van vs SPF-Van + *C. scindens* $P < 0.0001$). 2 days after the colonization, experimental colitis was induced by a 7-day treatment with DSS (2.5% in drinking water) followed by 3 days of drinking water (recovery period) (Fig. 2M). (**N**, **O**) Representative images (**N**) and quantification of EdU$^+$ cells per crypt (**O**) in the colon of unchallenged mice in (**A**) at the time of sacrifice. Scale bar = 50 µm (**M**, **N**). Graphs represent mean ± SEM. *n* refers to biological replicates. *P* values (exact values) were calculated using one-way ANOVA followed by Bonferroni's post hoc correction (**A**, **B**) or 2-tailed Student's t test (**F**, **G**, **H**, **I**, **K**, **L**).

 © The Author(s)

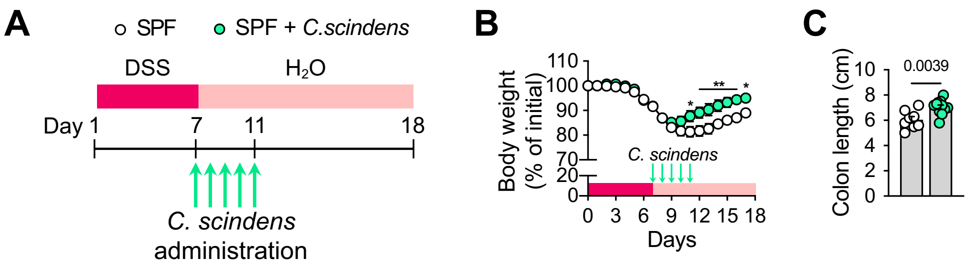

Figure EV3. Therapeutic effect of *C. scindens* administration in SPF mice.

(**A**) Workflow of the therapeutic DSS-induced acute experimental colitis experiment. 10-week-old male C57BL/6J mice were treated with DSS (2.5% in drinking water) for 7 days followed by a recovery phase. From the first day of the recovery period, mice were gavaged daily for 5 days with live *C. scindens* (10^8 CFU – SPF + *C. scindens* ($n = 11$)) or vehicle (PBS – SPF ($n = 9$)). Mouse body weight was monitored daily until one of the four experimental groups reached the initial body weight. (**B**) Percentage of body weight loss of mice in (**A**) during exposure to DSS and the recovery phase (SPF-Van vs SPF-Van + *C. scindens* day 11: *$P = 0.02$; day 12: **$P = 0.003$; day 13: **$P = 0.002$; day 14: **$P = 0.003$; day 15: **$P = 0.004$; day 16: **$P = 0.004$; day 17: *$P = 0.04$). (**C**) Colon length of mice in (**A**) at the end of the experiment. Graphs represent mean ± SEM. *n* refers to biological replicates. *P* values (exact values) were calculated using two-way ANOVA followed by Bonferroni's post hoc correction (**B**) or 2-tailed Student's t-test (**C**).

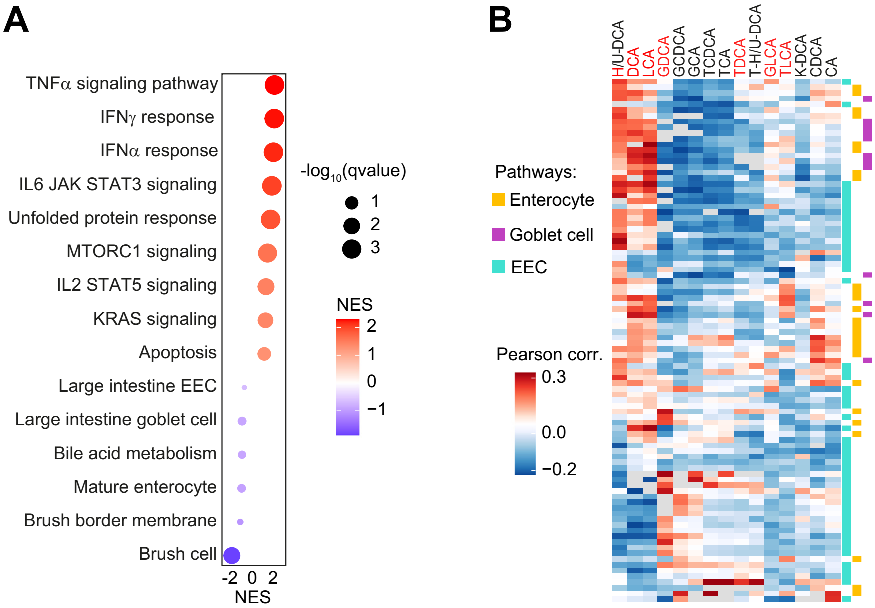

Figure EV4. 7α-dehydroxylated BAs are associated with intestinal cell differentiation in the colon of UC patients.

(**A**) Gene set enrichment analysis (GSEA) representing a selection of the most modulated biological processes in the colon of UC patients compared to non-IBD controls (publicly available dataset from (Lloyd-Price et al, 2019)), ordered by normalized enrichment score (NES). (**B**) Heatmap representing the correlation (Pearson correlation) between the abundance of different fecal BA species and the genes most significantly involved in biological processes of enterocytes, goblet cells and enteroendocrine cells (EEC) of colon in UC patients and non-IBD controls in (**A**). 7α-dehydroxylated BAs are highlighted in red.

 © The Author(s)
